# Correction: A musculoskeletal finite element model of rat knee joint for evaluating cartilage biomechanics during gait

**DOI:** 10.1371/journal.pcbi.1011025

**Published:** 2023-03-29

**Authors:** Gustavo A. Orozco, Kalle Karjalainen, Eng Kuan Moo, Lauri Stenroth, Petri Tanska, Jaqueline Lourdes Rios, Teemu V. Tuomainen, Mikko J. Nissi, Hanna Isaksson, Walter Herzog, Rami K. Korhonen

The Funding statement is incomplete. The complete Funding statement is as follows:

Academy of Finland (https://www.aka.fi/en; grant nos. 285909 [MJN], 324529 [RKK], 332915 [LS], 334773—under the frame of ERA PerMed [RKK]), Strategic Funding of the University of Eastern Finland [PT], Swedish Research Council (2019–00953—under the frame of ERA PerMed [HI]), European Regional Development Fund [MJN], Innovation Fund Denmark (9088-00006B - under the frame of ERA PerMed [LS]), Finnish Cultural Foundation (grant no. 191044 and no. 200059 [PT]), Marie Skłodowska-Curie Actions postdoctoral fellowship (project no. 890936 [EKM]), Maire Lisko Foundation [PT], Sigrid Juselius Foundation [RKK], Päivikki ja Sakari Sohlberg Foundation [GAO], Maud Kuistila Memorial Foundation [GAO], Saastamoinen Foundation [GAO], The Killam Foundation [WH], The Canada Research Chair Programme [WH] and The Canadian Institutes for Health Research [WH]. The funders had no role in study design, data collection and analysis, decision to publish, or preparation of the manuscript.
