# Supplementary material for: A musculoskeletal finite element model of rat knee joint for evaluating cartilage biomechanics during gait
Source: PLoS Comput Biol. 2022 Jun 3;18(6):e1009398. doi: 10.1371/journal.pcbi.1009398 (PMC9166403; doi:10.1371/journal.pcbi.1009398)
Supplement: S1 Supplementary material — Figure A. Distribution of joint reaction forces at lateral and medial as a function of stance. (a, b) Total joint reaction force, (c, d) the cartilage-cartilage, and (e, f) cartilage-menisci contact interfaces. Figure B. The effect of variations in the fibril network modulus on the average contact pressure, maximum principal strain, maximum principal stress, and fluid pressure in the contact area of the medial (a, c, e, and g) and lateral (b, d, f, and h) tibial cartilage surfaces during the stance phase of gait. Figure C. The effect of variations in the non-fibrillar matrix modulus on the average contact pressure, maximum principal strain, maximum principal stress, and fluid pressure in the contact area of the medial (a, c, e, and g) and lateral (b, d, f, and h) tibial cartilage surfaces during the stance phase of gait. Figure D. The effect of variations in the initial permeability on the average contact pressure, maximum principal strain, maximum principal stress, and fluid pressure in the contact area of the medial (a, c, e, and g) and lateral (b, d, f, and h) tibial cartilage surfaces during the stance phase of gait. Figure E. The effect of variations in the Poisson´s ratio of the non-fibrillar matrix on the average contact pressure, maximum principal strain, maximum principal stress, and fluid pressure in the contact area of the medial (a, c, e, and g) and lateral (b, d, f, and h) tibial cartilage surfaces during the stance phase of gait. Figure F. Comparisons of the effect of variations in the FRPE material properties on the maximum principal stress distributions in the tibial cartilage at 50% of the stance phase of gait (Lat: lateral: Med: medial). Figure G. Peak contact pressure, maximum principal strain, maximum principal stress, and fluid pressure in the contact area of the medial (a, c, e, and g) and lateral (b, d, f, and h) tibial cartilage surfaces during the stance phase of gait. (DOCX) [file pcbi.1009398.s001.docx]

*Supplementary material*

**A musculoskeletal finite element model of rat knee joint for evaluating cartilage biomechanics during gait**

*Gustavo A. Orozco^1,2^, Kalle Karjalainen^1^, Eng Kuan Moo^1,3^, Lauri Stenroth^1,4^, Petri Tanska^1^, Jaqueline Lourdes Rios^3,5^, Teemu V. Tuomainen^1^, Mikko J. Nissi^1^, Hanna Isaksson^2^, Walter Herzog^3^, Rami K. Korhonen^1^

*^1^Department of Applied Physics, University of Eastern Finland, Kuopio, Finland*

*Yliopistonranta 1, FI-70210 Kuopio, Finland*

*^2^Department* *of Biomedical Engineering, Lund University, Box 188, 221 00, Lund, Sweden*

*^3^Faculty of Kinesiology, Human Performance Laboratory, University of Calgary, 2500, University Drive NW, Calgary, Alberta, Canada T2N1N4*

*^4^Department of Biomedical Sciences, University of Copenhagen, Denmark*

*^5^* *Regenerative Medicine Center Utrecht, University Medical Center Utrecht, Utrecht, Netherlands*

**Keywords:** rat knee joint, articular cartilage, finite element model, degeneration, gait cycle, MRI

**Short title:** Rat knee joint computational model

**Corresponding author:**

*Gustavo A. Orozco

*Department of Applied Physics, University of Eastern Finland, Kuopio, Finland*

*Yliopistonranta 1, 70210 Kuopio, FI*

*Tel: +358 50 3485018*

[gustavo.orozco@uef.fi](mailto:gustavo.orozco@uef.fi)

**Results**

**Table A** - Fitted FRPE material parameters from indentation experiments on tibial cartilage.

| Sample | Average thickness (mm) | $E_{f}$ (MPa) | $E_{\mathrm{nf}}$ (MPa) | $k_{0}$ (10^-15^ m^4^/(Ns)) | *M* | R^2^ |
| --- | --- | --- | --- | --- | --- | --- |
| *S1* | 0.167 | 8.500 | 0.900 | 0.501 | 1.016 | 0.99 |
| *S2* | 0.080 | 2.230 | 1.005 | 7.401 | 1.511 | 0.94 |
| *S3* | 0.114 | 3.520 | 1.120 | 3.342 | 2.970 | 0.97 |
| *S4* | 0.120 | 0.500 | 0.510 | 0.691 | 1.493 | 0.92 |
| *S5* | 0.138 | 1.751 | 0.879 | 0.623 | 1.675 | 0.99 |
| *S6* | 0.113 | 2.283 | 0.594 | 7.221 | 1.329 | 0.99 |
| *mean* | **0.122** | **3.13** | **0.835** | **3.30** | **1.67** | **0.97** |
| *std* | 0.027 | 2.56 | 0.216 | 0.0030 | 0.62 | 0.03 |

$E_{f}$=fibril network modulus, $E_{\mathrm{nf}}$=nonfibrillar matrix modulus, $k_{0}$=initial permeability, *M* = exponential term for the strain-dependent permeability*.*


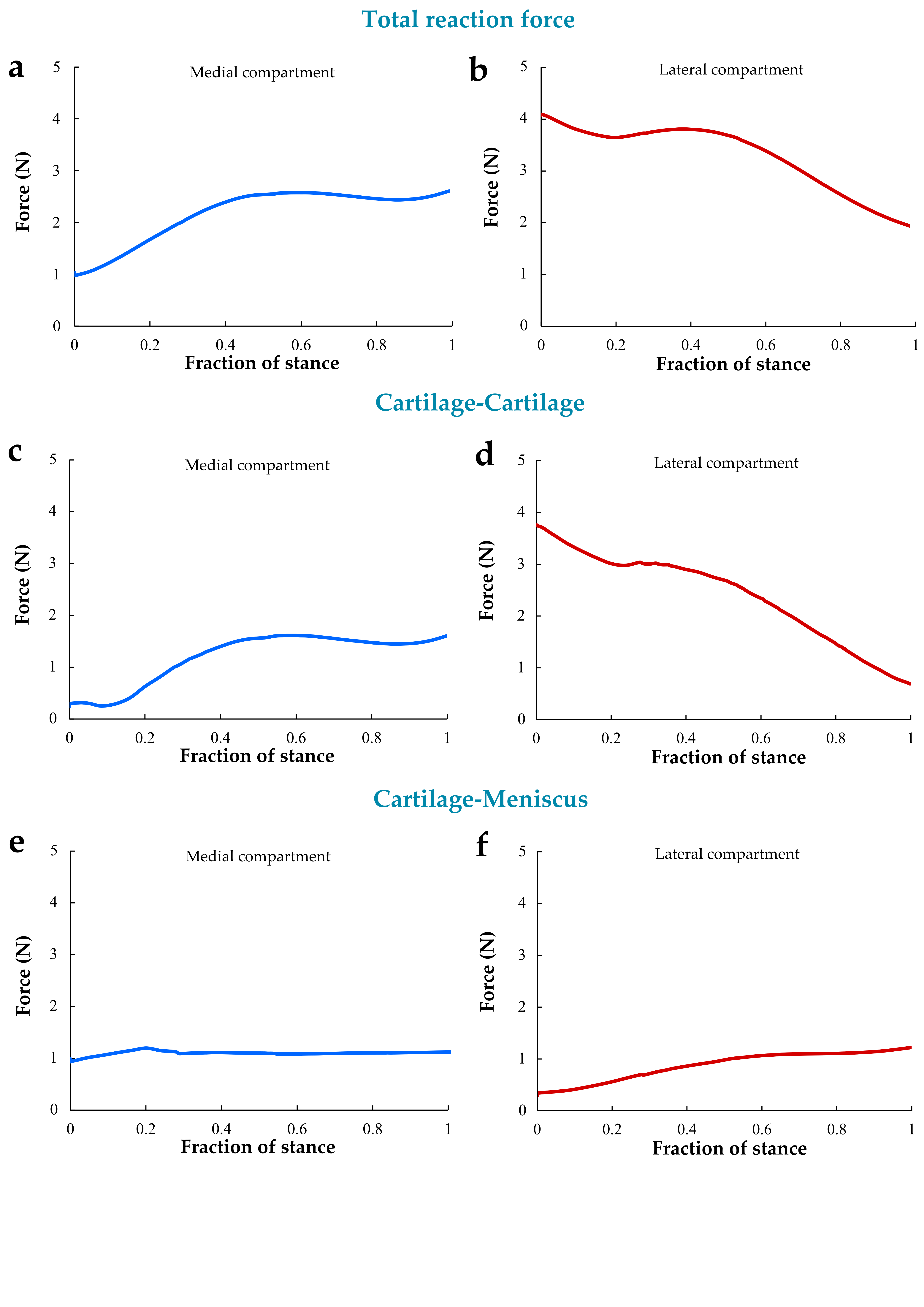


**Figure A.** Distribution of joint reaction forces at lateral and medial as a function of stance. (a, b) Total joint reaction force, (c, d) the cartilage-cartilage, and (e, f) cartilage-menisci contact interfaces.


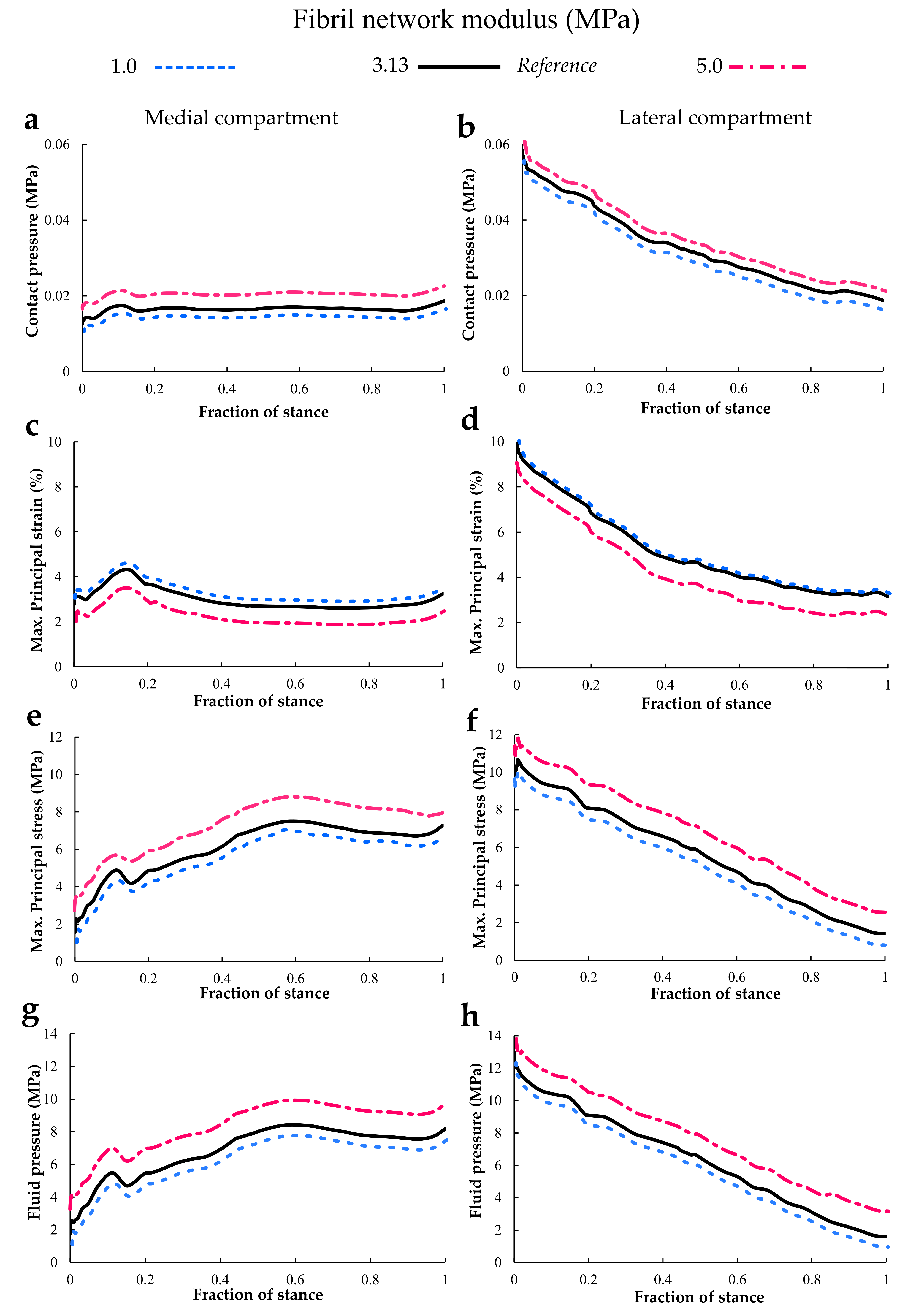


**Figure B.** The effect of variations in the fibril network modulus on the average contact pressure, maximum principal strain, maximum principal stress, and fluid pressure in the contact area of the medial (a, c, e, and g) and lateral (b, d, f, and h) tibial cartilage surfaces during the stance phase of gait.


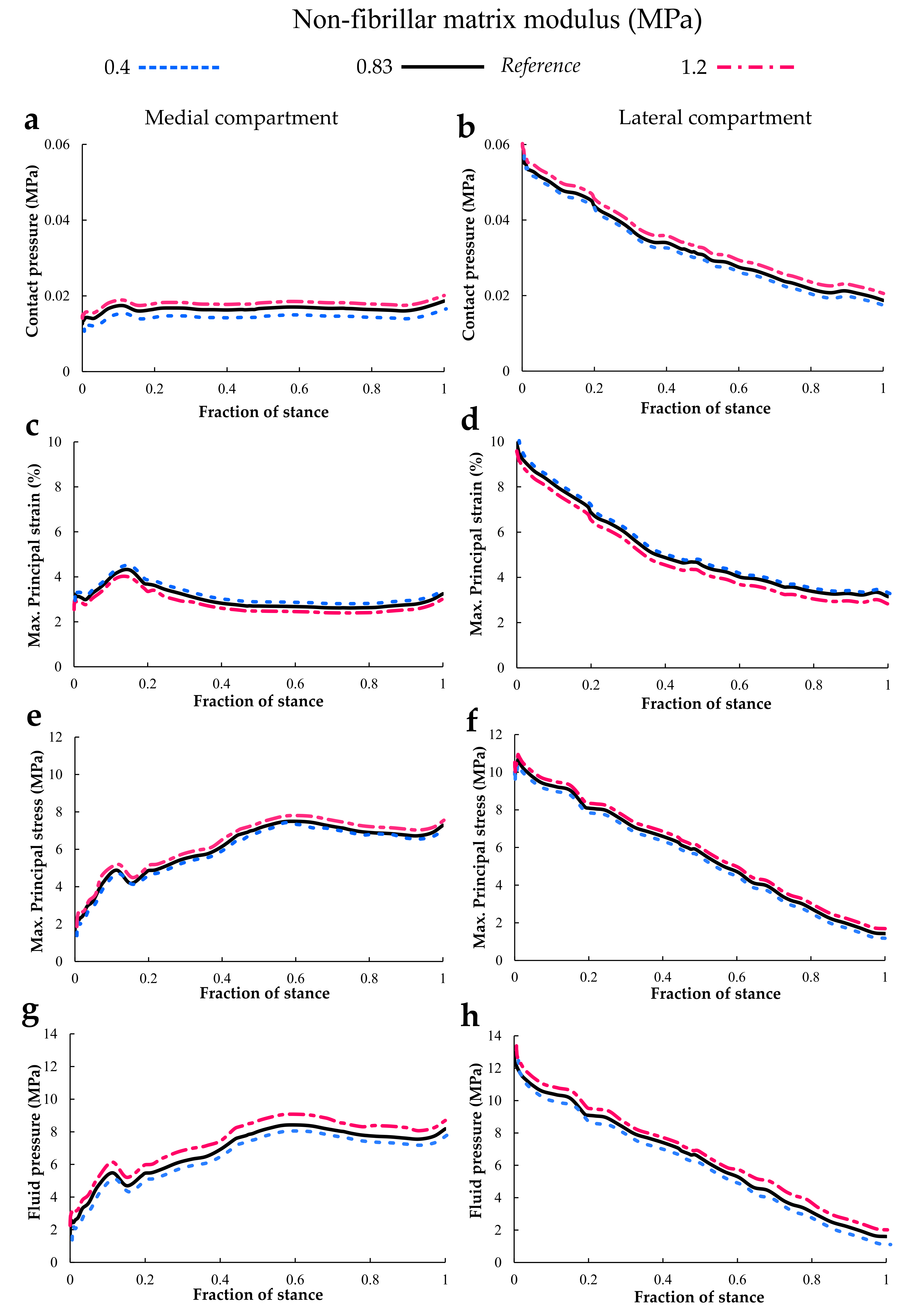


**Figure C.** The effect of variations in the non-fibrillar matrix modulus on the average contact pressure, maximum principal strain, maximum principal stress, and fluid pressure in the contact area of the medial (a, c, e, and g) and lateral (b, d, f, and h) tibial cartilage surfaces during the stance phase of gait.


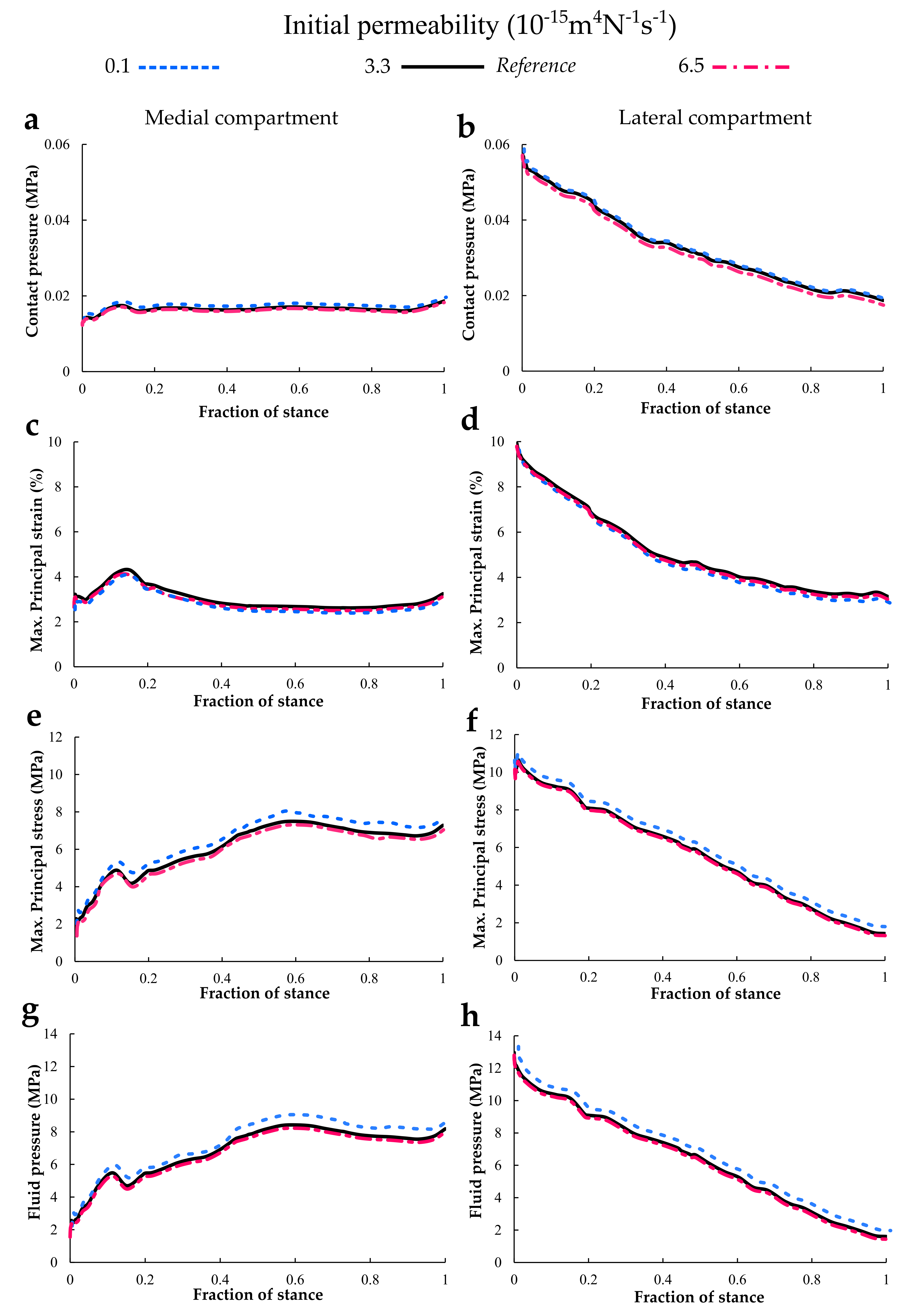


**Figure D.** The effect of variations in the initial permeability on the average contact pressure, maximum principal strain, maximum principal stress, and fluid pressure in the contact area of the medial (a, c, e, and g) and lateral (b, d, f, and h) tibial cartilage surfaces during the stance phase of gait.


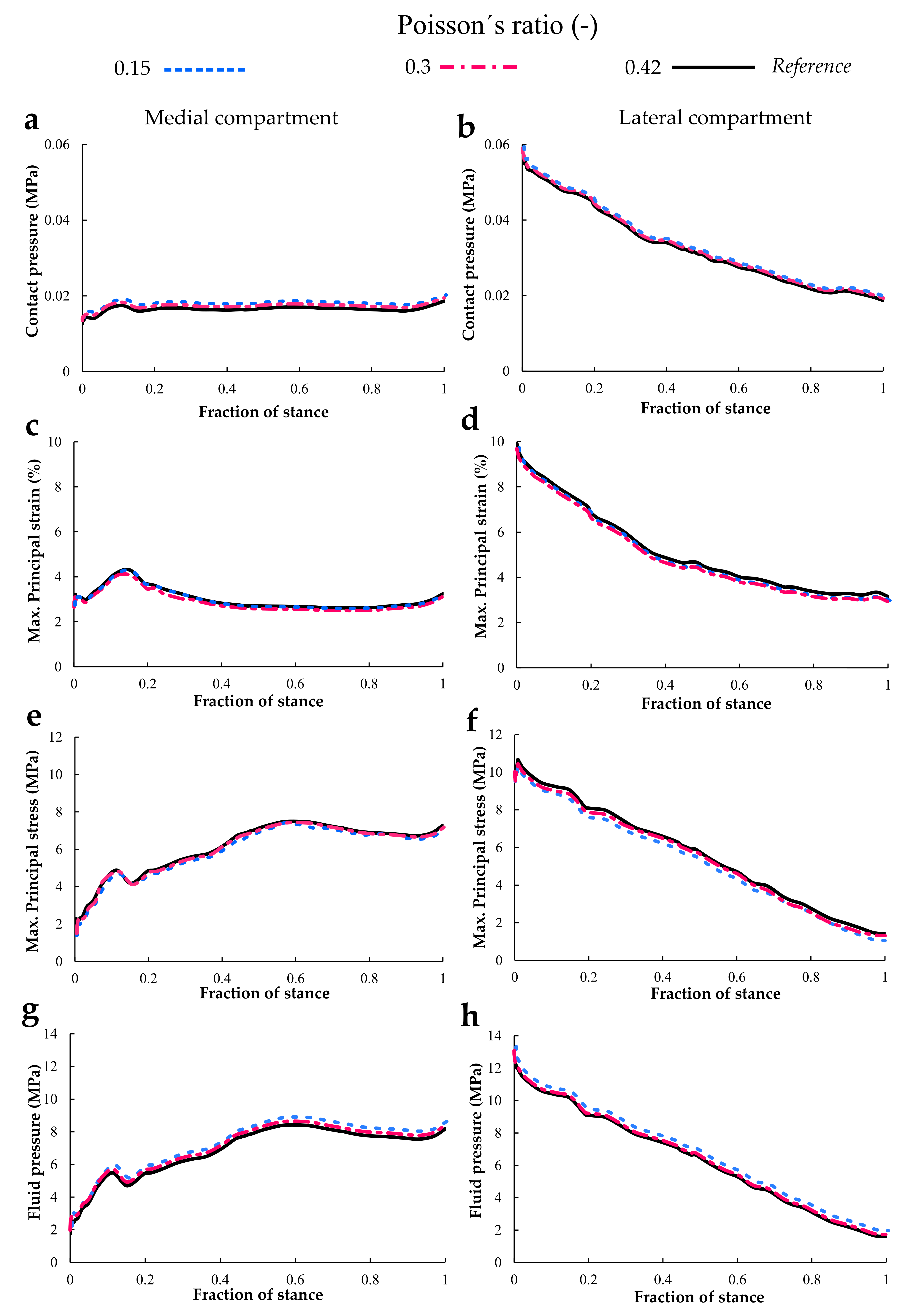


**Figure E.** The effect of variations in the Poisson´s ratio of the non-fibrillar matrix on the average contact pressure, maximum principal strain, maximum principal stress, and fluid pressure in the contact area of the medial (a, c, e, and g) and lateral (b, d, f, and h) tibial cartilage surfaces during the stance phase of gait.


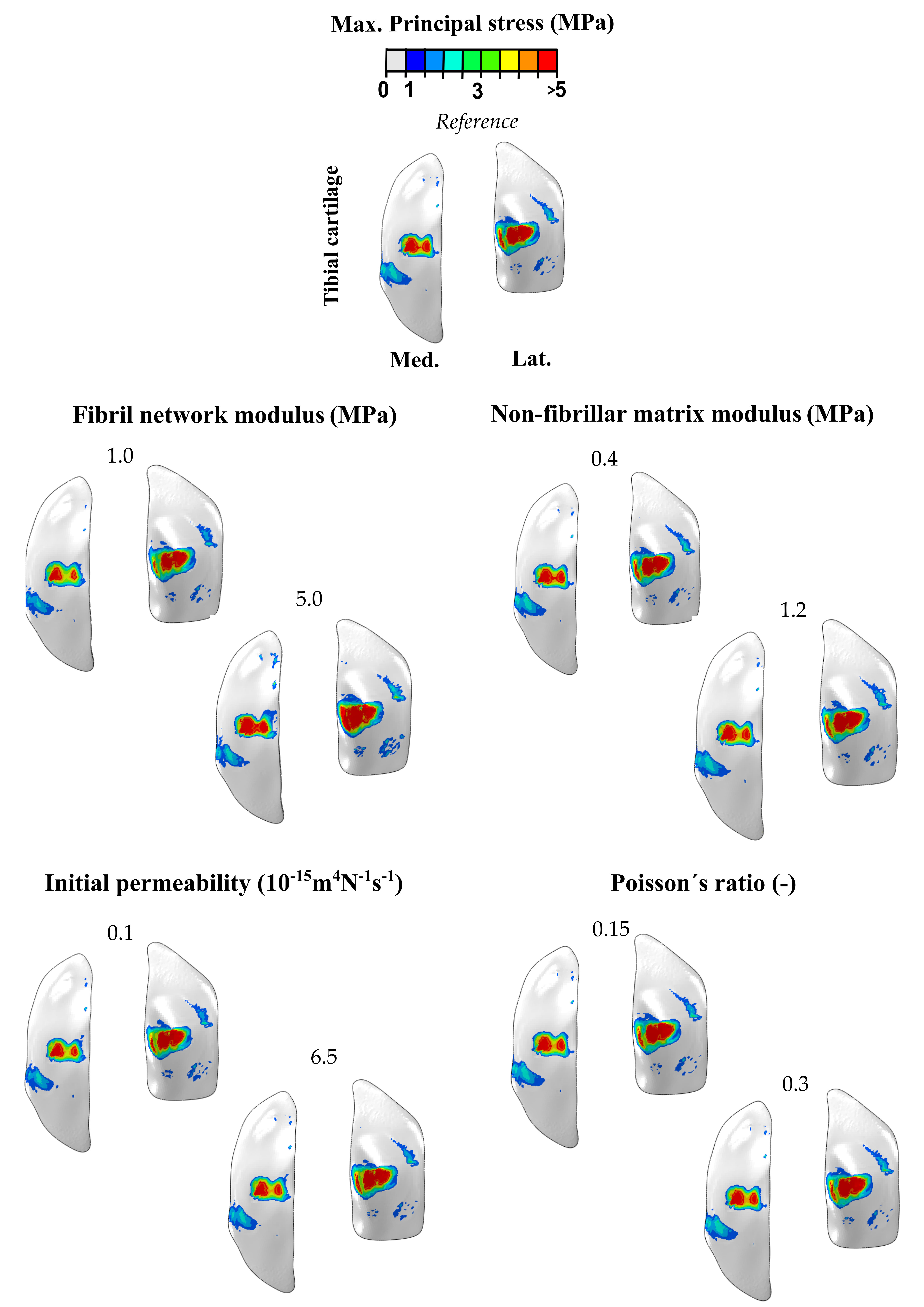


**Figure F.** Comparisons of the effect of variations in the FRPE material properties on the maximum principal stress distributions in the tibial cartilage at 50% of the stance phase of gait (Lat: lateral: Med: medial).


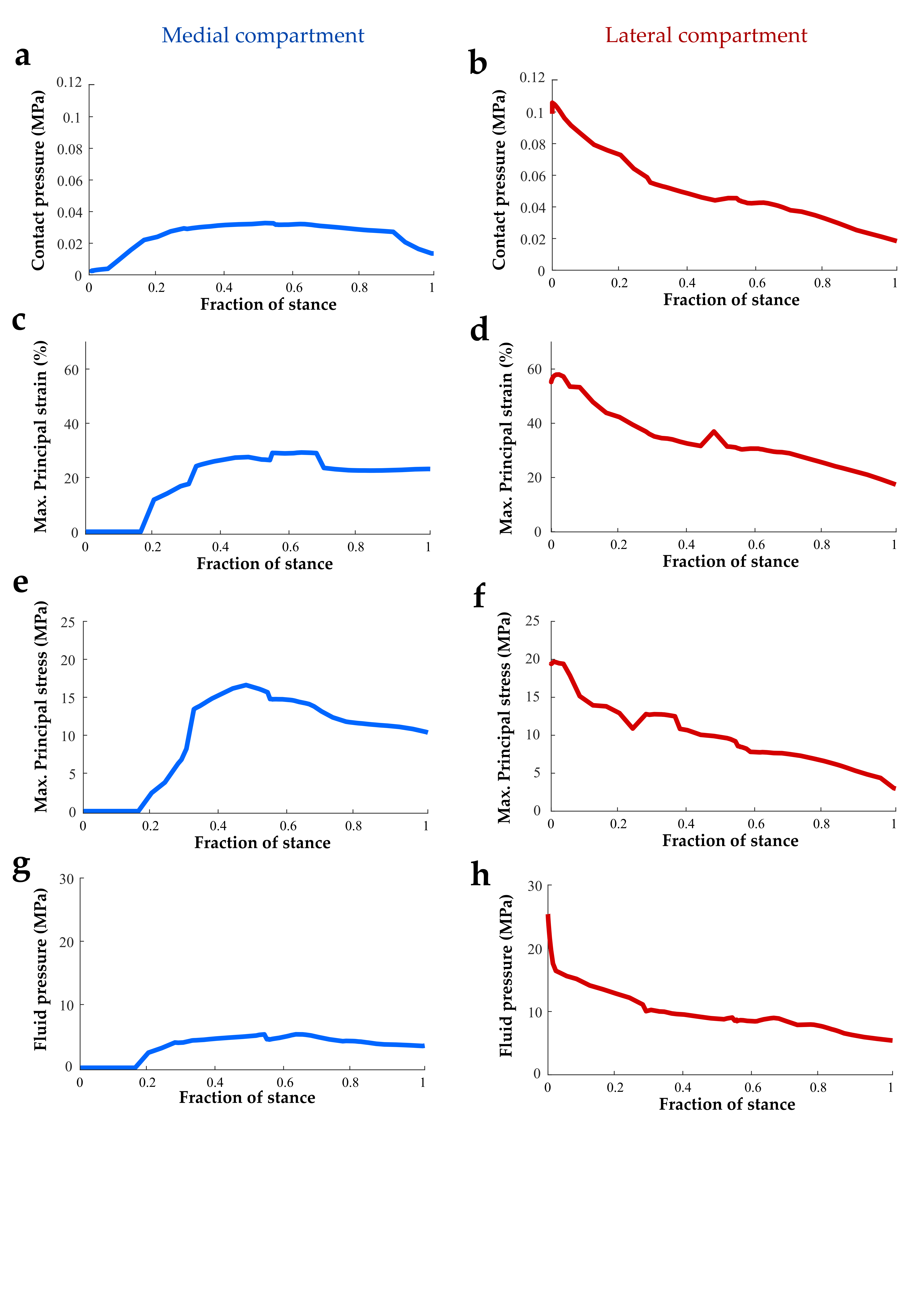


**Figure G.** Peak contact pressure, maximum principal strain, maximum principal stress, and fluid pressure in the contact area of the medial (a, c, e, and g) and lateral (b, d, f, and h) tibial cartilage surfaces during the stance phase of gait.
